# New Criteria on Oscillatory and Asymptotic Behavior of Third-Order Nonlinear Dynamic Equations with Nonlinear Neutral Terms

**DOI:** 10.3390/e23020227

**Published:** 2021-02-15

**Authors:** Said R. Grace, Jehad Alzabut, Abdullah Özbekler

**Affiliations:** 1 Department of Engineering Mathematics, Faculty of Engineering, Cairo University, Giza 12221, Egypt; saidgrace@yahoo.com; 2 Department of Mathematics and General Sciences, Prince Sultan University, Riyadh 11586, Saudi Arabia; 3 Department of Mathematics, Atilim University, Incek, Ankara 06830, Turkey; aozbekler@gmail.com or

**Keywords:** oscillation, asymptotic behavior, nonlinear neutral term, third-order dynamic equations

## Abstract

In the paper, we provide sufficient conditions for the oscillatory and asymptotic behavior of a new type of third-order nonlinear dynamic equations with mixed nonlinear neutral terms. Our theorems not only improve and extend existing theorems in the literature but also provide a new approach as far as the nonlinear neutral terms are concerned. The main results are illustrated by some particular examples.

## 1. Introduction

Let T be an arbitrary time scale with supT=∞ and $T_{0}=\left[\varsigma_{0},\infty \right)\cap T$. In the paper, we consider the following third-order dynamic equation of the form

(1) $${\left(a\left(\varsigma \right){\left[y^{\Delta \Delta }\left(\varsigma \right)\right]}^{\alpha }\right)}^{\Delta }+q\left(\varsigma \right)x^{\gamma }\left(\tau \left(\varsigma \right)\right)=0;\;\varsigma \in T_{0},$$

where

(2) $$y\left(\varsigma \right)=x\left(\varsigma \right)+\omega p\left(\varsigma \right)x^{\beta }\left(\delta \left(\varsigma \right)\right)$$

with ω=±1. Throughout the paper, we assume that

(i) α, β and γ are the ratios of two positive odd integers with α≥1;
(ii) *a*, *p* and $q\in C_{rd}\left(T_{0},R_{+}\right)$;
(iii) τ, $\delta \in C_{rd}\left(T_{0},T\right)$ such that τ(ς), δ(ς)≤ς, and δ is invertible with δ(ς)→∞ as ς→∞;
(iv) $h^{*}(\delta^{-1}\left(\tau \left(\varsigma \right)\right)\leq \varsigma$ and $h^{*}\to \infty$ as ς→∞.

Let

(3) $$\lim_{\varsigma \to \infty }A\left(\varsigma ,\varsigma_{0}\right)=\infty ,$$

where

$$A\left(u,v\right):=\int_{v}^{u}a^{-1/\alpha }\left(s\right)\Delta s.$$

We define the solution *x* of Equation (1) as a continuous function on $\left[T_{x},\infty \right)$ which satisfies Equation (1) on $[T_{x},\infty$), $T_{x}\geq \varsigma_{0}$. We only consider those solutions *x* of Equation (1) satisfying

$$\sup\left\{|x\left(\varsigma \right)|:\varsigma \geq T\right\}>0\;\mathrm{for all}\;T\geq T_{x}.$$

A solution *x* of Equation (1) is said to be oscillatory if there exists a sequence $\left\{\xi_{n}\right\}$ such that $x(\xi_{n})=0$ with $\lim_{n\to \infty }\xi_{n}=0$, and otherwise it is non-oscillatory. If all solutions of Equation (1) are oscillatory, then it is said to be oscillatory.

The oscillatory behavior of dynamic equations on time scales has become a very popular subject for many researchers, and thus it has been widely developed. For recent investigations regarding the systematic treatments of oscillations of solutions for second-order dynamic equations, we refer to [1,2,3,4] and the references cited therein. On the other hand, it has been realized that the oscillations of nonlinear third-order neutral equations contribute to many disciplines, including mechanical oscillation, earthquake structures, clinical applications, frequency measurements and harmonic oscillators that involve symmetrical properties; see, for instance, the pioneering monographs of [5,6]. Inspired by these extensive applications, many authors have paid more attention to studying the oscillatory behavior of third-order difference and differential equations. We review some relevant results for the sake of completeness.

In [7], the authors studied asymptotic properties of the third-order neutral differential equation of the form

(4) $${\left[a\left(\varsigma \right){\left({\left[x\left(\varsigma \right)\pm p\left(\varsigma \right)x\left(\delta \left(\varsigma \right)\right)\right]}^{\prime \prime }\right)}^{\gamma }\right]}^{\prime }+q\left(\varsigma \right)x^{\gamma }\left(\tau \left(\varsigma \right)\right)=0;\;\varsigma \geq \varsigma_{0},$$

where a,q,p are positive functions, γ>0 is a quotient of odd positive integers and τ(ς)≤ς,δ(ς)≤ς. Sufficient conditions are established which ensure that all nonoscillatory solutions of Equation (4) converge to zero. Very recently in [8], the following third-order nonlinear neutral differential equation was considered.

(5) $${\left[a\left(\varsigma \right){\left(z^{\prime \prime }\left(\varsigma \right)\right)}^{\gamma }\right]}^{\prime }+q\left(\varsigma \right)f\left(x\left(\tau \left(\varsigma \right)\right)\right)=0;\;\varsigma \geq \varsigma_{0}>0,$$

where z(ς)=x(ς)+p(ς)x(δ(ς)) and γ is a ratio of odd positive integers. New oscillation criteria have been introduced under the two cases $\int_{\varsigma_{0}}^{\infty }a^{-1/\gamma }\left(s\right)ds<\infty$ and $\int_{\varsigma_{0}}^{\infty }a^{-1/\gamma }\left(s\right)ds=\infty$. For more significant results, the reader can consult the papers [6,9,10,11,12,13].

After exploring the above-mentioned literature and to the best of authors’ knowledge, there have been no results published with regard to the oscillation and asymptotic behavior of third-order, nonlinear neutral differential equations as far as the nonlinear neutral terms are concerned. In this paper, we recover this case and obtain some sufficient conditions which assure that Equation (1) is either oscillatory or any of its solutions converge to zero. Evidently, it is shown that the existing literature does not guarantee such behavior for the solutions of Equation (1). Several examples are presented to validate and support the proposed suppositions.

## 2. Main Results

We state the following handy definition.

**Definition** **1.** *[9] Taylor monomials are the functions $h_{n}:T\times T\to R$, $n\in N_{0}=\left\{0,1,2,\ldots \right\}$, which are recursively defined as*

$$h_{0}\left(\mu ,s\right):=1$$

*and*

$$h_{n+1}\left(\mu ,s\right)=\int_{s}^{\mu }h_{n}\left(\tau ,s\right)▵\tau \;\left(n\in N_{0}\right)$$

*for μ,s∈T. It follows that $h_{1}\left(\mu ,s\right)=\mu -s$ on any time scale.*

One should observe that finding $h_{n}$ for n≥2 is not an easy task in general. For a particular time scale such as T=R or T=Z, we can easily find the functions $h_{n}$. Indeed, we have

(6) $$h_{n}\left(\mu ,s\right)=\frac{{\left(\mu -s\right)}^{n}}{n!}\;\left(\mu ,s\in R\right)\;\mathrm{and}\;h_{n}\left(\mu ,s\right)=\frac{{\left(\mu -s\right)}^{\bar{n}}}{n!}\;\left(\mu ,s\in Z\right),$$

where

$$\mu^{\bar{n}}:=\prod_{j=0}^{n-1}\left(\mu +j\right).$$

We present the main results of this paper in four parts.

### 2.1. Equation (1) When ω=1 and β≤1

The following result deals with the oscillation and asymptotic behavior of (1) with a sub-linear neutral term.

**Theorem** **1.** *Let conditions (i)–(iv), and (3) hold and assume that*

(7) $$\lim_{\varsigma \to \infty }p\left(\varsigma \right)=0.$$

*If*

(8) $$\underset{\varsigma \to \infty }{lim sup}\int_{\tau (\varsigma )}^{\varsigma }q\left(u\right){\left(\int_{\varsigma_{1}}^{\tau (u)}A\left(s,\varsigma_{1}\right)\Delta s\right)}^{\gamma }\Delta u=\infty \;\left(\gamma \leq \alpha \right)$$

*for $\varsigma_{1}\in \left[\varsigma_{0},\infty \right)$, then Equation (1) is oscillatory or every solution of it converges to zero.*

**Proof.**  Assume that x(ς)>0 is a (i.e., non-oscillatory) solution of Equation (1) and that

$$\lim_{\varsigma \to \infty }x\left(\varsigma \right)\neq 0,$$

x(τ(ς))>0, and $x\left(\delta \left(\varsigma \right)\right)>0$ for $\varsigma \geq \varsigma_{1}\geq \varsigma_{0}$. Equation (1) implies that

(9) $${\left(a\left(\varsigma \right){\left[y^{\Delta \Delta }\left(\varsigma \right)\right]}^{\alpha }\right)}^{\Delta }=-q\left(\varsigma \right)x^{\gamma }\left(\tau \left(\varsigma \right)\right).$$

Hence $a\left(\varsigma \right){\left[y^{\Delta \Delta }\left(\varsigma \right)\right]}^{\alpha }$ is non-increasing for $\varsigma \in {\left[\varsigma_{1},\infty \right)}_{T}$ and thus is of one sign. We claim that there exists a $\varsigma_{2}\geq \varsigma_{1}$ such that $y^{\Delta \Delta }\left(\varsigma \right)>0$ for $\varsigma \geq \varsigma_{2}$. Let the contrary hold. Then, we have

$$a\left(\varsigma \right){\left[y^{\Delta \Delta }\left(\varsigma \right)\right]}^{\alpha }\leq -b$$

for $\varsigma \geq \varsigma_{2}$ and for some positive constant *b*. Integrating this inequality from $\varsigma_{2}$ to ς and using condition (3) we obtain

$$y^{\Delta }\left(\varsigma \right)\leq y^{\Delta }\left(\varsigma_{2}\right)-b\int_{\varsigma_{2}}^{\varsigma }a^{-1/\alpha }\left(s\right)\Delta s\to -\infty \;as\;\varsigma \to \infty ,$$

which is a contradiction, hence we have $y^{\Delta \Delta }\left(\varsigma \right)>0$ for $\varsigma \geq \varsigma_{2}$.

To this end, we shall distinguish the following two cases for $\varsigma \geq \varsigma_{2}$:

I. y(ς)>0 and $y^{\Delta }\left(\varsigma \right)>0$;
II. y(ς)>0 and $y^{\Delta }\left(\varsigma \right)<0$.

Case I. (2) implies that

$$x\left(\varsigma \right)=y\left(\varsigma \right)-p\left(\varsigma \right)x^{\beta }\left(\delta \left(\varsigma \right)\right)\geq y\left(\varsigma \right)\left(1-\frac{p(\varsigma )}{y^{1-\beta }\left(\varsigma \right)}\right).$$

Since y(ς) is non-decreasing, we have

$$x\left(\varsigma \right)\geq \left[1-p\left(\varsigma \right)c_{*}^{\beta -1}\right]y\left(\varsigma \right)$$

for some positive constant $c_{*}>0$ such that $y\geq c_{*}$. This implies that there exists a constant c∈(0,1) such that

(10) x(ς)≥cy(ς).

Thus, we have

(11) $${\left(a\left(\varsigma \right){\left[y^{\Delta \Delta }\left(\varsigma \right)\right]}^{\alpha }\right)}^{\Delta }\leq -c^{\gamma }q\left(\varsigma \right)y^{\gamma }\left(\tau \left(\varsigma \right)\right).$$

Since $a\left(\varsigma \right){\left[y^{\Delta \Delta }\left(\varsigma \right)\right]}^{\alpha }$ is a non-increasing function, we conclude that $y^{\Delta \Delta }\left(\varsigma \right)>0$ and $y^{\Delta }\left(\varsigma \right)>0$ for $\varsigma \geq \varsigma_{2}$. It is clear to see that

$$y^{\Delta }\left(\varsigma \right)\geq A^{-1/\alpha }\left(\varsigma ,\varsigma_{1}\right)a^{1/\alpha }\left(\varsigma \right)y^{\Delta \Delta }\left(\varsigma \right).$$

Integration of both sides of the inequality above from $\varsigma_{1}$ to ς gives

$$y\left(\varsigma \right)\geq a^{1/\alpha }\left(\varsigma \right)y^{\Delta \Delta }\left(\varsigma \right)\int_{\varsigma_{1}}^{\varsigma }A\left(s,\varsigma_{1}\right)\Delta s.$$

Using the last inequality, (11) turns out to be

(12) $$-W^{\Delta }\left(\varsigma \right)\geq c^{\gamma }q\left(\varsigma \right)W^{\gamma /\alpha }\left(\tau \left(\varsigma \right)\right){\left(\int_{\varsigma_{1}}^{\tau (\varsigma )}A\left(s,\varsigma_{1}\right)\Delta s\right)}^{\gamma },$$

where $W\left(\varsigma \right)=a\left(\varsigma \right){\left[y^{\Delta \Delta }\left(\varsigma \right)\right]}^{\alpha }$. Now, integration of both sides of inequality (12) from τ(ς) to ς gives

$$\begin{array}{cc} W(\tau (\varsigma )) & \geq -W(\varsigma )+W(\tau (\varsigma ))\\ \; & \geq c^{\gamma }W^{\gamma /\alpha }\left(\tau \left(\varsigma \right)\right)\int_{\tau (\varsigma )}^{\varsigma }q\left(u\right){\left(\int_{\varsigma_{1}}^{\tau (u)}A\left(s,\varsigma_{1}\right)\Delta s\right)}^{\gamma }\Delta u. \end{array}$$

Through multiplying both sides of the resulting inequality by $W^{-\gamma /\alpha }\left(\tau \left(\varsigma \right)\right)$, we obtain

(13) $$W^{1-\gamma /\alpha }\left(\tau \left(\varsigma \right)\right)\geq c^{\gamma }\int_{\tau (\varsigma )}^{\varsigma }q\left(u\right){\left(\int_{\varsigma_{1}}^{\tau (u)}A\left(s,\varsigma_{1}\right)\Delta s\right)}^{\gamma }\Delta u.$$

By taking the limit supremum of both sides of (13) as ς→∞, we get

$$\underset{\varsigma \to \infty }{lim sup}\int_{\tau (\varsigma )}^{\varsigma }q\left(u\right){\left(\int_{\varsigma_{1}}^{\tau (u)}A\left(s,\varsigma_{1}\right)\Delta s\right)}^{\gamma }\Delta u<\infty ,$$

which contradicts with condition (8) of the theorem.

Case II. By condition (7), it is easy to see that any solution converges to zero. This completes the proof. □

We present the following illustrative example.

**Example** **1.** *Let T=R and consider the neutral functional differential equation:*

(14) $${\left(\varsigma^{3}{\left[y^{\prime \prime }\left(\varsigma \right)\right]}^{3}\right)}^{\prime }+x\left(\varsigma /2\right)=0,$$

*where*

$$y\left(\varsigma \right)=x\left(\varsigma \right)+\varsigma^{-1}x^{1/3}\left(\varsigma /2\right).$$

*Here we have $a\left(\varsigma \right)=\varsigma^{3}$, p(ς)=1/ς, α=3, τ(ς)=δ(ς)=ς/2. It is easy to check that the conditions of Theorem 1 are satisfied, and hence every solution of Equation (14) is either oscillatory or converges to zero.*

### 2.2. Equation (1) When ω=1 and β≥1

The following result is related to the oscillatory and asymptotic behavior of (1) with a super-linear neutral term.

**Theorem** **2.** *Let conditions (i)–(iv), and (3) hold and assume that*

(15) $$\lim_{\varsigma \to \infty }p\left(\varsigma \right){\left\{h_{1}\left(\varsigma ,\varsigma_{1}\right)A\left(\varsigma ,\varsigma_{1}\right)\right\}}^{\beta -1}=0.$$

*If condition (8) holds for $\varsigma_{1}\in {\left[\varsigma_{0},\infty \right)}_{T}$, then Equation (1) is either oscillatory or every solution of it converges to zero.*

**Proof.**  Let x(ς)>0 be a (i.e., non-oscillatory) solution of Equation (1) and

$$\lim_{\varsigma \to \infty }x\left(\varsigma \right)\neq 0,$$

x(τ(ς))>0, and $x\left(\delta \left(\varsigma \right)\right)>0$ for $\varsigma \geq \varsigma_{1}\geq \varsigma_{0}$. As in the proof of Theorem 1, we see that there exists a $\varsigma_{2}\geq \varsigma_{1}$ such that $y^{\Delta \Delta }\left(\varsigma \right)>0$. We shall distinguish the following two cases for $\varsigma \geq \varsigma_{2}$:

I. y(ς)>0 and $y^{\Delta }\left(\varsigma \right)>0$;
II. y(ς)>0 and $y^{\Delta }\left(\varsigma \right)<0$.

Case I: Since $a\left(\varsigma \right){\left[y^{\Delta \Delta }\left(\varsigma \right)\right]}^{\alpha }$ is a non-increasing function, $a\left(\varsigma \right){\left[y^{\Delta \Delta }\left(\varsigma \right)\right]}^{\alpha }\leq d_{1}$ for some positive constant $d_{1}>0$, and hence we have $y^{\Delta }\left(\varsigma \right)\leq d_{2}A\left(\varsigma ,\varsigma_{2}\right)$ for any $d_{2}>0$. Since $A(\varsigma ,\varsigma_{1})$ is an increasing function in ς, we see that there exists a constant d>0 such that $y\left(\varsigma \right)\leq dh_{1}\left(\varsigma ,\varsigma_{2}\right)A\left(\varsigma ,\varsigma_{1}\right)$ for $\varsigma \geq \varsigma_{2}$ ($\varsigma_{2}\geq \varsigma_{1}$). Using (2), we have

$$x\left(\varsigma \right)=y\left(\varsigma \right)-p\left(\varsigma \right)x^{\beta }\left(\delta \left(\varsigma \right)\right)\geq y\left(\varsigma \right)\left(1-p\left(\varsigma \right){\left\{dh_{1}\left(\varsigma ,\varsigma_{2}\right)A\left(\varsigma ,\varsigma_{1}\right)\right\}}^{\beta -1}\right)$$

for $\varsigma \geq \varsigma_{2}$. By condition (15), we have x(ς)≥cy(ς) for some constant c∈(0,1). The rest of the proof is left to the reader since it is analogous to that of Theorem 1. □

**Example** **2.** *Consider the difference equation (i.e., T=Z)*

(16) $$\Delta \left(n^{3}{\left[\Delta^{2}y\left(n\right)\right]}^{3}\right)+x\left(n/2\right)=0,$$

*where*

$$y\left(n\right)=x\left(n\right)+n^{-3}x^{3}\left(n/2\right).$$

*Here we have $a\left(n\right)=n^{3}$, $p\left(n\right)=n^{-3}$, α=3 and τ(n)=δ(n)=n/2. It can be verified that the conditions of Theorem 2 are satisfied. Thus we conclude that Equation (16) is either oscillatory or every solution of it converges to zero.*

For convenience, we let

$$B\left(\varsigma \right)=\frac{1}{p(\delta^{-1}\left(\varsigma \right))}\left[1-c{\left\{p(\delta^{-1}\left(\delta^{-1}\left(\varsigma \right)\right))\right\}}^{-1/\beta }\right]\geq 0$$

for any constant c>0 and $\varsigma \in {\left[\varsigma_{0},\infty \right)}_{T}$. Further, we assume

(iii)${}^{*}$ $\tau ,\delta \in C_{rd}\left(T_{0},T\right)$ such that $\xi \left(\varsigma \right)=\delta^{-1}\left(\tau \left(\left(\varsigma \right)\right)\right)\leq \varsigma$, δ(ς)≥ς, δ(ς) is non-decreasing and invertible, ξ(ς) is non-decreasing and
  $$\lim_{\varsigma \to \infty }\xi \left(\varsigma \right)=\infty .$$

**Theorem** **3.** *Let conditions* (i), (ii), (iii)${}^{*}$ and (3) hold. If

(17) $$\underset{\varsigma \to \infty }{lim sup}\int_{\xi (\varsigma )}^{\varsigma }q\left(u\right){\left[B\left(\tau \left(u\right)\right)\right]}^{\gamma /\beta }{\left(\int_{\varsigma_{1}}^{\xi (u)}A\left(\xi \left(s\right),\varsigma_{1}\right)\Delta s\right)}^{\gamma /\beta }\Delta u=\infty \;\left(\gamma \leq \alpha \beta \right)$$

*for $\varsigma_{1}\in {\left[\varsigma_{0},\infty \right)}_{T}$, then Equation (1) is either oscillatory or every solution of it converges to zero.*

**Proof.**  Let x(ς)>0 be a (non-oscillatory) solution of Equation (1) and

$$\lim_{\varsigma \to \infty }x\left(\varsigma \right)\neq 0,$$

x(τ(ς))>0 and x(δ(ς))>0 for $\varsigma \geq \varsigma_{1}$ and $\varsigma_{1}\in {\left[\varsigma_{0},\infty \right)}_{T}$. Equation (1) implies that

(18) $${\left(a\left(\varsigma \right){\left[y^{\Delta \Delta }\left(\varsigma \right)\right]}^{\alpha }\right)}^{\Delta }=-q\left(\varsigma \right)x^{\gamma }\left(\tau \left(\varsigma \right)\right)\leq 0.$$

As in the proof of Theorem 1, we see that there exists a $\varsigma_{2}\geq \varsigma_{1}$ such that

(I.) $a\left(\varsigma \right){\left[y^{\Delta \Delta }\left(\varsigma \right)\right]}^{\alpha }>0$;
(II.) $a\left(\varsigma \right){\left[y^{\Delta \Delta }\left(\varsigma \right)\right]}^{\alpha }<0$ and $y^{\Delta }\left(\varsigma \right)>0$ for $\varsigma \geq \varsigma_{2}$.

For cases (I) and (II), we have $y^{\Delta }\left(\varsigma \right)>0$ for $\varsigma \geq \varsigma_{2}$. Thus, there exists a positive constant $c_{1}>0$ and a $\varsigma_{3}\geq \varsigma_{2}$ such that $y\left(\delta^{-1}\left(\varsigma \right)\right)\geq c_{1}$ and

$$0<y^{1/\alpha -1}\left(\delta^{-1}\left(\varsigma \right)\right)\leq c_{1}^{1/\alpha -1}:=c.$$

Clearly, we have y(ς)≥x(ς) and

$$x^{\beta }\left(\delta \left(\varsigma \right)\right)=\frac{1}{p(\varsigma )}\left[y(\varsigma )-x(\varsigma )\right]\leq \frac{y(\varsigma )}{p(\varsigma )}.$$

It is easy to see that

$$x\left(\delta^{-1}\left(\varsigma \right)\right)\leq {\left[p\left(\delta^{-1}\left(\delta^{-1}\left(\varsigma \right)\right)\right)\right]}^{-1/\beta }{\left[y\left(\delta^{-1}\left(\delta^{-1}\left(\varsigma \right)\right)\right)\right]}^{1/\beta }$$

and

$$\begin{array}{cc} x^{\beta }\left(\varsigma \right) & =\frac{1}{p(\delta^{-1}\left(\varsigma \right))}\left[y\left(\delta^{-1}\left(\varsigma \right)\right)-x\left(\delta^{-1}\left(\varsigma \right)\right)\right]\\ \; & \geq \frac{1}{p(\delta^{-1}\left(\varsigma \right))}\left\{y\left(\delta^{-1}\left(\varsigma \right)\right)-{\left[p\left(\delta^{-1}\left(\delta^{-1}\left(\varsigma \right)\right)\right)\right]}^{-1/\beta }{\left[y\left(\delta^{-1}\left(\delta^{-1}\left(\varsigma \right)\right)\right)\right]}^{1/\beta }\right\}. \end{array}$$

Using the facts that *y* is non-decreasing, δ(ς)≥ς and $\delta^{-1}\left(\delta^{-1}\left(\varsigma \right)\right)\leq \delta^{-1}\left(\varsigma \right)$, we have

$${\left[y\left(\delta^{-1}\left(\delta^{-1}\left(\varsigma \right)\right)\right)\right]}^{1/\beta }\leq {\left[y\left(\delta^{-1}\left(\varsigma \right)\right)\right]}^{1/\beta }$$

and

$$\begin{array}{cc} x^{\beta }\left(\varsigma \right) & \geq \frac{1}{p(\delta^{-1}\left(\varsigma \right))}\left\{1-\frac{{\left[y(\delta^{-1}\left(\varsigma \right))\right]}^{1/\alpha -1}}{{\left[p(\delta^{-1}\left(\delta^{-1}\left(\varsigma \right)\right))\right]}^{1/\alpha }}\right\}y\left(\delta^{-1}\left(\varsigma \right)\right)\\ \; & \geq \frac{1}{p(\delta^{-1}\left(\varsigma \right))}\left\{1-c{\left[p(\delta^{-1}\left(\delta^{-1}\left(\varsigma \right)\right))\right]}^{-1/\alpha }\right\}y\left(\delta^{-1}\left(\varsigma \right)\right)\\ \; & \geq B\left(\varsigma \right)y(\delta^{-1}\left(\varsigma \right)) \end{array}$$

which implies

$$x^{\beta }\left(\tau \left(\varsigma \right)\right)\geq B\left(\tau \left(\varsigma \right)\right)y\left(\delta^{-1}\left(\tau \left(\varsigma \right)\right)\right)=B\left(\tau \left(\varsigma \right)\right)y\left(\xi \left(\varsigma \right)\right).$$

Using (9) and (10) turns out to be

$${\left(a\left(\varsigma \right){\left[y^{\Delta \Delta }\left(\varsigma \right)\right]}^{\alpha }\right)}^{\Delta }\leq -q\left(\varsigma \right){\left[B\left(\tau \left(\varsigma \right)\right)\right]}^{\gamma /\beta }{\left[y\left(\xi \left(\varsigma \right)\right)\right]}^{\gamma /\beta }.$$

The rest of the proof is left to the reader, since it is similar to that of the above case. □

We have the following example.

**Example** **3.** *Let T=R and consider the functional neutral differential equations*

(19) $${\left(\varsigma^{-6}{\left[y^{\prime \prime }\left(\varsigma \right)\right]}^{3}\right)}^{\prime }+\varsigma^{-10}x^{3}\left(\tau \left(\varsigma \right)\right)=0$$

*and*

(20) $${\left(e^{\varsigma }{\left[y^{\prime \prime }\left(\varsigma \right)\right]}^{3}\right)}^{\prime }+e^{\varsigma }x^{3}\left(\tau \left(\varsigma \right)\right)=0,$$

*where $y\left(\varsigma \right)=x\left(\varsigma \right)+tx^{3}\left(2\varsigma \right)$. Choose τ(ς)=3ς/2, τ(ς)=ς or τ(ς)=2ς/3; that is, Equations (19) or (20) is either advanced, ordinary or retarded. Since δ(ς)=2ς, we have ξ(ς)=3ς/4, ξ(ς)=ς/2 or ξ(ς)=ς/3, respectively. Clearly*

$$B\left(\varsigma \right)=2\left(\varsigma^{1/3}-4^{1/3}\right)\varsigma^{-4/3}\geq 0$$

*for ς≥4. It can be simply seen that condition (17) is satisfied, and hence we conclude that Equations (19) and (20) are either oscillatory or every solution of them tends to zero.*

### 2.3. Equation (1) When p(ς)=0

In this subsection, we obtain a new oscillation criterion for the equation

(21) $${\left(a\left(\varsigma \right){\left[x^{\Delta \Delta }\left(\varsigma \right)\right]}^{\alpha }\right)}^{\Delta }+q\left(\varsigma \right)x^{\gamma }\left(\tau \left(\varsigma \right)\right)=0.$$

**Theorem** **4.** *Let conditions (i)–(iv) and (3) hold. If*

(22) $$\underset{\varsigma \to \infty }{lim sup}\int_{\tau (\varsigma )}^{\varsigma }q\left(u\right){\left(\int_{\varsigma_{1}}^{\tau (u)}A\left(s,\varsigma_{1}\right)\Delta s\right)}^{\gamma }\Delta u=\infty \;\left(\gamma \leq \alpha \right)$$

*for $\varsigma_{1}\in {\left[\varsigma_{0},\infty \right)}_{T}$ and we assume that there exists a non-decreasing function $\zeta \left(\varsigma \right)\in C_{rd}\left(T_{0},T\right)$ such that ζ>ς, η(ς):=ζ(ζ(ς))>ς, η(τ(ς))≤ς and*

$$\lim_{\varsigma \to \infty }\eta \left(\tau \left(\varsigma \right)\right)=\infty ,$$

*and that*

(23) $$\underset{\varsigma \to \infty }{lim sup}\int_{\eta (\tau (\varsigma ))}^{\varsigma }q\left(u\right){\left(\int_{u}^{\zeta (\tau (u))}a^{-1/\alpha }\left(s\right)h_{1}\left(\zeta \left(s\right),s\right)\Delta s\right)}^{\gamma }\Delta u>\left\{\begin{array}{c} 1\;if\;\gamma =\alpha \\ 0\;if\;\gamma <\alpha \end{array}\right.,$$

*then Equation (21) is oscillatory.*

**Proof.**  Let x(ς)>0 be a (i.e., non-oscillatory) solution of Equation (21) and x(τ(ς))>0 for $\varsigma \geq \varsigma_{1}\geq \varsigma_{0}$. As in the proof of Theorem 1, we see that there exists a $\varsigma_{2}\geq \varsigma_{1}$ such that $x^{\Delta \Delta }\left(\varsigma \right)>0$ for $\varsigma \geq \varsigma_{2}$.

We shall examine the situation under two cases.

(I.) x(ς)>0 and $x^{\Delta }\left(\varsigma \right)>0$;
(II.) x(ς)>0 and $x^{\Delta }\left(\varsigma \right)<0$ for $\varsigma \geq \varsigma_{2}$.

By following the analogous steps as in the proof of Theorem 1 for case (I), we get a contradiction.

Case (II): It is easy to see that

$$\begin{array}{cc} -x^{\Delta }\left(\varsigma \right) & \geq x^{\Delta }\left(\zeta \left(\varsigma \right)\right)-x^{\Delta }\left(\varsigma \right)\\ \; & =\int_{\varsigma }^{\zeta (\varsigma )}x^{\Delta \Delta }\left(s\right)\Delta s\\ \; & \geq a^{-1/\alpha }\left(\varsigma \right)h_{1}\left(\zeta \left(\varsigma \right),\varsigma \right)a^{1/\alpha }\left(\zeta \left(\varsigma \right)\right)x^{\Delta \Delta }\left(\zeta \left(\varsigma \right)\right). \end{array}$$

Integrating the last inequality, we get

$$x\left(\varsigma \right)\geq a^{1/\alpha }\left(\eta \left(\varsigma \right)\right)x^{\Delta \Delta }\left(\eta \left(\varsigma \right)\right)\int_{\varsigma }^{\zeta (\varsigma )}a^{-1/\alpha }\left(s\right)h_{1}\left(\zeta \left(s\right),s\right)\Delta s$$

or

$$x\left(\tau \left(\varsigma \right)\right)\geq {\left[a\left(\eta \left(\tau \left(\varsigma \right)\right)\right)\right]}^{1/\alpha }x^{\Delta \Delta }\left(\eta \left(\tau \left(\varsigma \right)\right)\right)\int_{\varsigma }^{\zeta (\tau (\varsigma ))}a^{-1/\alpha }\left(s\right)h_{1}\left(\zeta \left(s\right),s\right)\Delta s.$$

Using the inequality above in Equation (21), we have

$$\begin{array}{cc} {\left(a\left(\varsigma \right){\left[x^{\Delta \Delta }\left(\varsigma \right)\right]}^{\alpha }\right)}^{\Delta }+q\left(\varsigma \right){\left\{{\left[a\left(\eta \left(\tau \left(\varsigma \right)\right)\right)\right]}^{1/\alpha }x^{\Delta \Delta }\left(\eta \left(\tau \left(\varsigma \right)\right)\right)\right\}}^{\gamma } & \\ \times {\left(\int_{\varsigma }^{\zeta (\tau (\varsigma ))}a^{-1/\alpha }\left(s\right)h_{1}\left(\zeta \left(s\right),s\right)\Delta s\right)}^{\gamma }\leq 0 \end{array}$$

or

$$X^{\Delta }\left(\varsigma \right)+q\left(\varsigma \right){\left[X\left(\eta \left(\tau \left(\varsigma \right)\right)\right)\right]}^{\gamma /\alpha }{\left(\int_{\varsigma }^{\zeta (\tau (\varsigma ))}a^{-1/\alpha }\left(s\right)h_{1}\left(\zeta \left(s\right),s\right)\Delta s\right)}^{\gamma }\leq 0,$$

where $X\left(\varsigma \right)=a\left(\varsigma \right){\left[x^{\Delta \Delta }\left(\varsigma \right)\right]}^{\alpha }$. The rest of the proof is omitted since it is similar to that of Theorem 1. □

**Example** **4.** *Let T=R and consider the equation*

(24) $${\left(\varsigma {\left[x^{\prime \prime }\left(\varsigma \right)\right]}^{3}\right)}^{\prime }+x\left(4\varsigma /9\right)=0.$$

*Here we have a(ς)=ς, α=3, τ(ς)=4ς/9. Let ϱ(ς)=3ς/2, η(ς)=9ς/4 and so η(τ(ς))=ς/2. It can be simply verified that the conditions of Theorem 4 are satisfied. Thus, Equation (24) is oscillatory.*

### 2.4. Equation (1) When ω=−1

Define

(25) $$Q\left(\varsigma \right):=q\left(\varsigma \right){\left[p\left(\delta^{-1}\left(\tau \left(\varsigma \right)\right)\right)\right]}^{-\gamma /\beta }.$$

In this context, we have the following result.

**Theorem** **5.** Let conditions (i)–(iv) and (3) hold and assume that there exists a continuous function ξ(ς) with $h^{*}\left(\varsigma \right)\leq \xi \left(\varsigma \right)\leq \varsigma$, $\varsigma \geq \varsigma_{0}$, and

$$\lim_{\varsigma \to \infty }\xi \left(\varsigma \right)=\infty .$$

*If condition (18) and*

(26) $$\underset{\varsigma \to \infty }{lim sup}\int_{\xi (\varsigma )}^{\varsigma }Q\left(s\right){\left[h_{1}\left(h^{*}\left(s\right),\varsigma_{1}\right)\right]}^{\gamma /\beta }{\left(\left[\xi \left(s\right)-h^{*}\left(s\right)\right][a{\left(\xi \left(s\right)\right]}^{-1/\alpha }\right)}^{\gamma /\beta }\Delta s=\infty$$

*hold for γ≤αβ and $\varsigma_{1}\in {\left[\varsigma_{0},\infty \right)}_{T}$, then Equation (1) is either oscillatory or every solution of it converges to zero, where the function Q(ς) is defined in (25).*

**Proof.**  Let x(ς)>0 be a (i.e., non-oscillatory) solution of Equation (1) and

$$\lim_{\varsigma \to \infty }x\left(\varsigma \right)\neq 0,$$

x(τ(ς))>0, and $x\left(\delta \left(\varsigma \right)\right)>0$ for $\varsigma \geq \varsigma_{1}$, $\varsigma_{1}\in {\left[\varsigma_{0},\infty \right)}_{T}$. Equation (1) yields that

$${\left(a\left(\varsigma \right){\left[y^{\Delta \Delta }\left(\varsigma \right)\right]}^{\alpha }\right)}^{\Delta }=-q\left(\varsigma \right)x^{\gamma }\left(\tau \left(\varsigma \right)\right)\leq 0.$$

Hence $a\left(\varsigma \right){\left[y^{\Delta \Delta }\left(\varsigma \right)\right]}^{\alpha }$ is non-increasing and is of constant sign. As in the proof of Theorem 1, there exists a $\varsigma_{2}\geq \varsigma_{1}$ such that $y^{\Delta \Delta }\left(\varsigma \right)>0$ for $\varsigma \geq \varsigma_{2}$.

To this end, we examine the cases:

(I) y(ς)>0 and $y^{\Delta }\left(\varsigma \right)>0$;
(II) y(ς)>0 and $y^{\Delta }\left(\varsigma \right)<0$;
(III) y(ς)<0 and $y^{\Delta }\left(\varsigma \right)>0$;
(IV) y(ς)<0 and $y^{\Delta }\left(\varsigma \right)<0$.

Case (I): (2) implies that x(ς)≥y(ς). Thus, we have

(27) $${\left(a\left(\varsigma \right){\left[y^{\Delta \Delta }\left(\varsigma \right)\right]}^{\alpha }\right)}^{\Delta }\leq -q\left(\varsigma \right)y^{\gamma }\left(\tau \left(\varsigma \right)\right).$$

Since $y^{\Delta \Delta }\left(\varsigma \right)>0$ and $y^{\Delta }\left(\varsigma \right)>0$ for $\varsigma \geq \varsigma_{2}$, we get a contradiction by following the proof of Theorem 1 (Case I).

Case (II): This case is excluded.

Next, we consider Case (III) and Case (IV), when y(ς)<0 for $\varsigma \geq \varsigma_{2}$. Let

$$z\left(\varsigma \right)=-y\left(\varsigma \right)=-x\left(\varsigma \right)+p\left(\varsigma \right)x^{\beta }\left(\delta \left(\varsigma \right)\right)\leq p\left(\varsigma \right)x^{\beta }\left(\delta \left(\varsigma \right)\right).$$

Then we have

$$x\left(\delta \left(\varsigma \right)\right)\geq {\left(\frac{z(\varsigma )}{p(\varsigma )}\right)}^{1/\beta }$$

or

$$x\left(\varsigma \right)\geq {\left(\frac{z\left(\delta^{-1}\left(\varsigma \right)\right)}{p\left(\delta^{-1}\left(\varsigma \right)\right)}\right)}^{1/\beta }.$$

Hence, we have

(28) $$\begin{array}{cc} {\left(a\left(\varsigma \right){\left[z^{\Delta \Delta }\left(\varsigma \right)\right]}^{\alpha }\right)}^{\Delta } & =q\left(\varsigma \right)x^{\gamma }\left(\tau \left(\varsigma \right)\right)\\ & \geq q\left(\varsigma \right)\left[p\left(\delta^{-1}\left(\tau \left(\varsigma \right)\right)\right)\right){]}^{-\gamma /\beta }{\left[z\left(\delta^{-1}\left(\tau \left(\varsigma \right)\right)\right)\right]}^{\gamma /\beta }\\ & =Q\left(\varsigma \right){\left[z\left(h^{*}\left(\varsigma \right)\right)\right]}^{\gamma /\beta }. \end{array}$$

Case (III): In this case we have $z^{\Delta \Delta }\left(\varsigma \right)<0$ and so $z^{\Delta }\left(\varsigma \right)\leq 0$. However, this contradicts with Condition (3).

Case (IV): In this case we have $z^{\Delta \Delta }\left(\varsigma \right)<0$ and so $z^{\Delta }\left(\varsigma \right)\geq 0$. It is easy to see that

$$z\left(h^{*}\left(\varsigma \right)\right)\geq h_{1}\left(h^{*}\left(\varsigma \right),\varsigma_{1}\right)z^{\Delta }\left(h^{*}\left(\varsigma \right)\right).$$

Using the last inequality, (28) turns out that

$${\left(a\left(\varsigma \right){\left[z^{\Delta \Delta }\left(\varsigma \right)\right]}^{\alpha }\right)}^{\Delta }\geq Q\left(\varsigma \right)[z{\left(h\left(\varsigma \right)\right]}^{\gamma /\beta }\geq Q\left(\varsigma \right){\left[h_{1}\left(h^{*}\left(\varsigma \right),\varsigma_{1}\right)\right]}^{\gamma /\beta }{\left[z^{\Delta }\left(h^{*}\left(\varsigma \right)\right)\right]}^{\gamma /\beta },$$

i.e.,

(29) $${\left(a\left(\varsigma \right){\left[Z^{\Delta }\left(\varsigma \right)\right]}^{\alpha }\right)}^{\Delta }\geq Q\left(\varsigma \right){\left[h_{1}\left(h^{*}\left(\varsigma \right),\varsigma_{1}\right)\right]}^{\gamma /\beta }{\left[Z\left(h^{*}\left(\varsigma \right)\right)\right]}^{\gamma /\beta },$$

where $Z\left(\varsigma \right)=z^{\Delta }\left(\varsigma \right)>0$. We see that

$$Z\left(u\right)-Z\left(v\right)\geq \left(v-u\right)\left(-Z^{\Delta }\left(v\right)\right)$$

for $v\geq u\geq \varsigma_{1}$. Setting $u=h^{*}\left(\varsigma \right)$ and v=ξ(ς) we have

(30) $$Z\left(h\left(\varsigma \right)\right)\geq \left[\xi \left(\varsigma \right)-h^{*}\left(\varsigma \right)\right]\left(-Z^{\Delta }(\xi \left(\varsigma \right)\right).$$

Using (29) and (30), we get

$$\begin{array}{cc} \; & {\left(a\left(\varsigma \right){\left[Z^{\Delta }\left(\varsigma \right)\right]}^{\alpha }\right)}^{\Delta }+Q\left(\varsigma \right){\left[h_{1}\left(h^{*}\left(\varsigma \right),\varsigma_{1}\right)\right]}^{\gamma /\beta }{\left[Z(h^{*}\left(\varsigma \right))\right]}^{\gamma /\beta }-X^{\Delta }\left(\varsigma \right)\\ \; & \;\geq Q\left(\varsigma \right){\left(\left[\xi \left(\varsigma \right)-h^{*}\left(\varsigma \right)\right]{\left[a\left(\xi \left(\varsigma \right)\right)\right]}^{-1/\alpha }\right)}^{\gamma /\beta }{\left[h_{1}\left(h^{*}\left(\varsigma \right),\varsigma_{1}\right)\right]}^{\gamma /\beta }{\left[X\left(\xi \left(\varsigma \right)\right)\right]}^{\gamma /\alpha \beta }, \end{array}$$

where $X\left(\varsigma \right)=-a\left(\varsigma \right){\left[Z^{\Delta }\left(\varsigma \right)\right]}^{\alpha }$. Due to the similarity to the above cases, the rest of the proof is left to the reader, and hence is omitted. □

**Example** **5.** *Let T=R and consider the functional differential equation*

(31) $${\left(\varsigma^{3}{\left({y\left(\varsigma \right))}^{\prime \prime }\right)}^{3}\right)}^{\prime }+x\left(\varsigma /2\right)=0,$$

*where*

$$y\left(\varsigma \right)=x\left(\varsigma \right)-x^{3}\left(\varsigma /2\right).$$

*Here we have $a\left(\varsigma \right)=\varsigma^{3}$, p(ς)=1, α=3, τ(ς)=δ(ς)=ς/2. It can be verified that all the conditions of Theorem 5 are satisfied, and hence Equation (31) is either oscillatory or every solution of it converges to zero.*

## 3. Conclusions

In this paper, we discussed the oscillatory behavior of a new type of third-order nonlinear dynamic equations with mixed nonlinear neutral terms. Particular emphasis was paid to the consideration of nonlinear neutral terms in the main equation, which has not been considered before. The proof of the main results was given based on the cases β≤1 and β≥1. It was demonstrated that the equations considered in the examples cannot be commented on by the results obtained in the literature [6,7,8,9,10,11,12,13]. Thus, the results of this paper complement and generalize somehow the existing results in the literature.

The results given in the paper can be generalized to the higher-order dynamic equations of the form

$${\left(a\left(\varsigma \right){\left[y^{\Delta^{\left(n-1\right)}}\left(\varsigma \right)\right]}^{\alpha }\right)}^{\Delta }+q\left(\varsigma \right)x^{\gamma }\left(\tau \left(\varsigma \right)\right)=0;\;n=4,5,\ldots$$

We leave this problem for further consideration in the future.

## Data Availability

Not applicable.
